# Synbiotic Supplements in the Prevention of Obesity and Obesity-Related Diseases

**DOI:** 10.3390/metabo12040313

**Published:** 2022-03-31

**Authors:** Emília Hijová

**Affiliations:** Center of Clinical and Preclinical Research (MEDIPARK), Department of Experimental Medicine, Faculty of Medicine, Pavol Jozef Šafárik University, 040 11 Košice, Slovakia; emilia.hijova@upjs.sk; Tel.: +421-552343463

**Keywords:** obesity, synbiotics, gut microbiota, type 2 diabetes mellitus, metabolic syndrome

## Abstract

Obesity and being overweight have reached incredible proportions worldwide and are one of the most common human health problems. The causes of obesity are multifactorial, including a complex interplay among genes, metabolism, diet, physical activity, and the environment. The intestinal microbiota has the ability to affect the host physiology for both benefit and damage, either directly or through microbial metabolites. The aim of this review is to discuss the mechanisms by which the intestinal microbiota could act as a key modifier of obesity and related metabolic abnormalities. The synbiotic components provide an optimal target for modulation of the intestinal microbiota and help reshape the metabolic profile in obese people. The development of novel functional foods containing synbiotic ingredients may present a support in the prevention of obesity as one of the risk factors for chronic diseases. Knowledge of the synbiotic mechanisms of action and the use of new functional foods supplemented with probiotics and prebiotics will facilitate the clinical application and development of individual health care strategies.

## 1. Introduction

Obesity has become one of the biggest general health challenges currently facing our society. Obesity and its complications are a significant burden. The prevalence of obesity is constantly rising worldwide, so it is considered an epidemic. Obesity and overweight, as risk factors for chronic diseases and their consequences associated with metabolic disorders and, more recently, as the factors influencing the severity of the new coronavirus disease (COVID-19), and at the same time, a serious independent disease in childhood and adulthood, represent serious health problems that require a multifactorial response [1]. According to the World Health Organization, in 2016, 39% of adults aged 18 years and over were overweight and 13% were obese [2,3]. The prevalence of overweight and obesity has also increased in children. Childhood obesity has risen exponentially over the last 25 years among infants and young children (aged 0–5 years) to 42 million in 2013 and is currently the most widespread eating disorder in the world [4]. Every sixth man aged 15 is overweight or obese [5]. The factors contributing to the obesity epidemic include the increased availability of energy-rich foods, increased sedentary activity, and possible involvement of the intestinal microbiota in host metabolism. Several authors described the new relationships between obesity and the intestinal microbiota, especially the change in the ratio of the two most dominant bacterial strains, Firmicutes and Bacteroidetes, in favour of Firmicutes in obese people [6,7,8].

Obesity is one of the controllable and “modifiable” risk factors for chronic diseases. To understand the origins and main factors supporting obesity and its physiological effects, detailed research is still needed to develop and effectively implement the potential therapeutic pathways to directly target metabolic pathology.

Modern research demonstrates the central role of the human gut microbiome in modulating human health and host metabolism. The human gut microbiome can act as a key player in obesity and related metabolic changes, including type 2 diabetes.

To develop strategies for the prevention of these intestinal microbiota imbalances, food companies have recently invested in finding new nutrients to manipulate and restore a healthy microbiota composition. Focusing on intestinal microbiota with synbiotics (probiotic supplements containing prebiotic components) is proving to be a promising intervention in a comprehensive nutritional approach to reduce obesity. Correction of obesity-induced disruption of the intestinal microbiota by synbiotics may be more effective than supplementation with probiotics alone because the prebiotic components of synbiotics support the growth and survival of positive bacteria in them.

## 2. Microbial Population and Obesity

Obesity is now declared the current global epidemic. The pathophysiology of obesity is multifactorial. The main factors that contribute to its development are unhealthy lifestyle, neuronal and hormonal mechanisms, as well as genetic and epigenetic factors contributing to the imbalance between energy intake and expenditure [9]. Obesity is a global epidemiologic syndrome characterised by the accumulation of fat, especially visceral fat.

Researchers are gaining a better understanding of the “normal” bacterial communities and physiology of the current intestinal microbiota through population research, such as the Human Microbiome Project [10]. Microbes detected in the human intestinal tract can be divided into three domains based on molecular phylogeny (i.e., similarities and differences in the sequence of 16S ribosomal ribonucleic acid [rRNA]): eukarya, bacteria, and archaea. Eukarya includes organisms whose cells contain complex structures surrounded by membranes, especially the nucleus. Bacteria are the predominant strains of the intestinal microbiota, and it is estimated that the adult intestinal tract contains approximately 500–1000 different bacterial species [11]. The dominant methanogenic species of the archaea in the human digestive system is *Methanobrevibacter smithii*. The most numerous faecal bacterial groups of both poor and obese subjects are the Firmicutes and Bacteriodetes strains. About 90% of all intestinal bacterial phylotypes belong to either the gram-positive Firmicutes (64%) or the gram-negative Bacteroidetes (23%) [12,13]. Other important strains are Proteobacteria, Actinobacteria, Verrucomicrobia, and Fusobacteria (Table 1).

The functions of microbiota are summarized as follows:

− The production of metabolites (the fermentation of complex carbohydrates leads to the production of short-chain fatty acids /SCFA/ and other lipogenic precursors that are necessary in many cellular processes and metabolic pathways, in the improvement of intestinal barrier function and regulation of the immune system and inflammatory reactions) [14];
− The metabolic organ (with enzymatic properties that improve or replace our own, such as the ability to degrade the resistant dietary or host-derived glycans that pass through the distal intestine, can control the bile acid metabolism and contribute to the induction/protection against metabolic endotoxemia) [15];
− The vitamin production (microbiota synthetizes the essential vitamins B12 and K that humans cannot produce, their dysregulation leads to metabolic pathologies, such as obesity and diabetes mellitus 2) [16];
− The influence on the epithelial homeostasis (microbiota suppors the epithelial integrity by influencing the epithelial cell turnover and modulating the mucus properties) [17];
− The development of the immune system (defense of the intestinal mucosa and the systemic immune system are modulated by microbiota, which leads to greater protection against infections and inflammatory diseases) [17];
− The impact on pathogen colonization (microbiota compete with pathogens for the attachement sites and nutrients, and produces the antimicrobials) [18].

Maintaining heterogenicity and stability within the gut microbiota community is fundamental for promoting host health. Alterations in diversity and microbiota community structure may influence host metabolism, which may contribute to the development of obesity. The first studies showing a link between the microbiome and an obese phenotype were carried out in germ-free (GF) mice, which were initially found to be sturdy against diet-induced obesity even under conditions of a high-fat diet [19]. Moreover, an obese phenotype was transmissible to these animals via faecal transplant from either Western diet-fed or genetically obese mice. The results showed that weight gain in these animals was higher than after the inoculation with wild-type microbes [7]. Similar results have been observed in colonisation studies with microbiota from pairs of mono and dizygotic human twins discordant for obesity. Specifically, microbial inoculation caused a progressively greater increase in fat mass and body weight in animals after intake of the microbes from the obese twin, despite no significant differences in energy intake between animal groups [20]. People with obesity have consistently demonstrated a decrease in diversity and richness in microbial populations, which can be inversed using weight loss intervention (nutrition low in fat and animal products and rich in fruit and vegetables) [21,22]. Microbial diversity has been connected to the metabolic function of gut microbiota, and low bacterial richness has been considered to be a risk factor for obesity and low-grade inflammation. An obesity-related host microbiome displays an enrichment in particular gene categories involved in carbohydrate and lipid metabolism and a decrease in enzymes involved in glucose and insulin signalling pathways [12,13]. Consistent microbiota finding have displayed the reductions in abundances of the families *Rikenellaceae* (phyla Firmicutes) and *Christensenellaceae* (phyla Firmicutes) as well as a decrease in the abundance of the genera *Bifidobacterium* (phyla Actinobacteria), *Oscillospira* (phyla Firmicutes) and *Akkermansia* (phyla Verrucomicrobia) [13,23,24]. Many of these depleted microbiota provide beneficial attributes to the host. *Bifidobacterium* is associated with elevated levels of short chain fatty acids (SCFAs), a decreased luminal lipoplysaccharide (LPS) and an improvement of intestinal barrier function [25]. *Akkermansia* is a mucin-degrading microbe that occupies the outer mucus layer of the intestinal barrier and is connected with a healthier metabolic status in obese humans. In obese people, it could be a potential prognostic marker for predicting the outcome of dietary intervention because an increased amount of *Akkermansia* in the gut after embarking on weight loss leads to improvement in glucose homeostasis, blood lipids, and body composition [26,27]. *Faecali prausnitzii*, a significant butyrate-producing microbe providing host protection against bacterial translocation, is considerably reduced in obesity, particularly in patients with diabetes mellitus [28]. Reducing weight in obese adults has been shown to have the opposite influence on microbiota composition and on an increase in the relative abundances of *Faecali prausnitzii*, *Akkermansia,* and *Christensenellaceae* [27,29,30]. Families *Prevotellaceae* (phyla Bacteroidetes), *Coriobacteriaceae* (phyla Actinobacteria), *Erysipelotrichaceae* (phyla Firmicutes)*, Alcaligenaceae* (phyla Proteobacteria), and genus *Roseburia* (phyla Firmicutes) are consistently reported in subjects with obesity and increased BMI (Body mass index) [31,32]. It is apparently demonstrated that a reduction in gut microbiome diversity occurs in obese subjects, but there are still many irresponsible questions about the exact microbial population of an obese gut microbiota. Whether it is more important to target microbiota composition at phyla or deeper levels such as genus and species remains open to discussion, and whether the absence, depletion, or presence of particular microbiota contributes to the emergence of obesity. The exact mechanism by which “obese microbiota” affects the development of obesity is still unfolding.

### Short-Chain Fatty Acids

The intestinal microbiota has the ability to affect host physiology for both benefit and damage, either directly or through microbial metabolites. The major microbial metabolites produced under anaerobic carbohydrate fermentation are short-chain fatty acids (SCFAs). SCFAs comprise of one to six carbons of that acetate (C2), propionate (C3), and butyrate (C4) are the most abundant (≥95%) [14,33]. SCFAs are transferred from the lumen through the apical membrane of the colonocyte into the colonocyte and then through the basolateral membrane of the colonocyte into portal blood. The SCFAs are either used by the intestinal epithelial cells for energy or are diffused to the portal vein from the intestinal lumen and are then taken up by various peripheral organs, where they act as substrates or signalling molecules with key G-protein-coupled receptors (GPR, now called Free Fatty Acid receptors—FFARs; GPR41/FFAR3, GPR43/FFAR2, GPR119, GPR109A), which are plentiful in adipocytes, intestinal immune cells, and epithelial cells to impact host energy homeostasis. SCFA production has been connected to a decrease in body weight and adiposity. These receptors are not stimulated alike by all SCFAs. Propionate mainly activates GPR41/FFAR3, butyrate activates GPR109A, while GPR43/FFAR2 and GPR119 can be activated by acetate, butyrate, and propionate at the same rates. The stimulation of the GPR41 and GPR 43 receptors raises the neuroendocrine secretion of peptide tyrosine-tyrosine (PYY), which inhibits gastric emptying and gut transit time, thereby inhibiting appetite and promoting glucagon-like peptide (GLP-1), the latter with a stimulatory influence on insulin secretion. These two intestinal hormones lessen gut motility, support satiety, and repress energy intake. SCFA-GPR41 and GPR43 interactions trigger the expression of leptin from adipocytes and impact the inflammatory responses that are severe for the development of obesity-related metabolic disorders such as insulin resistance, lipogenesis, and increased triglyceride stores. The leptin influence on the hypothalamus causes a decrease in food intake by inhibiting the release of neuropeptide Y (NPY) and promotes an increase in the host metabolic rate, consequently increasing energy expenditure. GPR43 coupling supports the creation of antimicrobial peptides RegIIIɣ and β-defensin and immunity-related cytokines such as interleukin (IL)-1, IL-6, IL-12 and IL-18 [15,31]. GPR109A enabled by butyrate, inhibits a colonic inflammation and carcinogenesis by promoting anti-inflammatory abilities in colonic macrophages and dendritic cells, which force the differentiation of regulatory T cells, IL-10 producing T cells and rise the secretion of IL-18 in gut epithelial cells [34].

Acetate, the most plentiful SCFA, is produced by gut bacteria from the genera *Lactobacillus*, *Bifidobacterium*, *Akkermansia*, *Bacteroides*, *Prevotella*, *Ruminococcus,* and *Streptococcus* [35]. Acetate is easily absorbed and moved to the liver to be used as an energy resource and as a substrate for the creation of cholesterol and long-chain fatty acids. Butyrate is created by the genera *Anaerostipes*, *Clostridium*, *Coprococcus*, *Dorea*, *Eubacterium*, *Faecalibacterium*, *Roseburia*, *Ruminococcus*, and the most plentiful producers appear to be the species *Faecalibacterium prausnitzii*, *Eubacterium rectale,* and *Roseburia intestinalis*. Butyrate is especially used by colonocytes as an energy source and is badly detected in the circulation. It exerts powerful anti-infective and anti-inflammatory abilities in the intestine and is able to prevent body weight gain without changing food intake and improve insulin sensitivity.

Butyrate supports energy expenditure and possibly decreases obesity through the increase in mitochondrial efficacy (activates AMP (adenosinmonophosphate)-activated protein kinase, increasing adenosine triphosphate (ATP) consumption and the release of PGC-1 (peroxisome proliferator-activated receptor gamma coactivator one) peroxisome proliferator-activated receptor gamma coactivator one), in connection with the upgrade regulation of the expression of genes involved in lipolysis and fatty acid oxidation. Butyrate may decrease energy intake by evoking a host anorexic reaction by increasing the plasma levels of glucagon-like peptide 1 (GLP-1), glucose dependent insulinotropic polypeptide (GIP) and peptide YY (PYY) [36].

Propionate is created by the genera *Phascolarctobacterium*, *Bacteroides*, *Dialister*, *Megasphaera*, *Veillonella*, *Coprococcus*, *Roseburia*, *Ruminococcus*, and *Salmonella* [35]. Propionate shows a protective effect by decreasing the risk of cancer development and is connected with the important systemic metabolic effects, it decreases serum cholesterol levels and liver lipogenesis, increases the levels of anorectic hormones PYY and GLP-1, and has the ability to encourage an intestinal-brain circuit via the receptor GPR41, thus leading to the support of intestinal gluconeogenesis (IGN) gene expression. Up-regulation of IGN by propionate decreases body weight gain and adiposity, independent of food supply [37,38].

Obesity in adults is caused by dysbiosis, which is also involved in the etiology of childhood obesity. Infant gut microbiota could be a biomarker to identify children who are at risk of becoming overweight or obese later in childhood. Human trials conducted to date demonstrate that obesity may be connected with a reduced bacterial diversity and shifts of gut bacteria at the phylum level; however, discrepancies exist in the directionality and suitability of the Firmicutes to Bacteroidetes ratio in obesity. It is likely that obesity-related intestinal dysbiosis has its origins in infancy, as early as 3–6 months after birth, at a time when the first colonisers of intestinal microbiota lay the basis for subsequent colonisation by anaerobes from the Bacteroidetes phylum. The structure of the gut microbiota at the age of 2 years can serve as a predictor of obesity at the age of 12 years [39]. This finding proposes that intestinal microbiota composition may be the earliest warning mark for detecting obesity risk. Obesity is the pre-stage that leads to metabolic syndrome and the main feature of this state is insulin resistance that much increases the risk for development of pre-diabetes, type 2 diabetes mellitus, cardiovascular diseases, and other diseases [40]. Obesity is one of the strongest predictors of diabetes mellitus, and a disorder of the intestinal microbiota in obesity may lead to a reduced creation of SCFAs, leading to an increase in factors of inflammation, influence on insulin secretion and islet B cell sensitivity, as well as insulin resistance. SCFA, mainly butyrate, supports the secretion of GLP-1, which inhibits the secretion of glucagon, inhibits gluconeogenesis in the liver, and optimises insulin sensitivity. SCFAs can prevent the low-grade inflammation caused by bacterial movement from the intestinal to mesenteric adipose tissue and blood [41]. These suggest that the increase in SCFAs, especially butyrate, is important for avoiding and controlling pre-diabetes and diabetes. Intestinal microbiota take part in colonisation resistance, which reinforces the mucosal barrier opposite to colonisation by pathogens and provides continuous stimulation of pathogen recognition receptors to increase the creation of mucins and antimicrobial peptides [42]. Contravention of the mucosal lining permits the translocation of toxins, resulting in metabolic endotoxaemia, increasing levels of lipopolysaccharides (LPS), and subsequent low-grade inflammation, autoimmunity, and oxidative stress. Gastrointestinal mucosal function involves several mechanisms, including the appropriate localisation and distribution of tight junction proteins, normal endocannabinoid system tone, and LPS detoxification by gut alkaline phosphatase [43]. LPS encourages inactive immune cells by binding with toll-like receptors (TLR), activating immune cells to release inflammatory cytokines, which supports insulin resistance caused by an endotoxin-induced inflammatory response. LPS acts with endocannabinoid receptors (eCB1), modulating intestinal permeability and LPS movement, increasing levels of circulating LPS and inducing metabolic endotoxemia [44].

It is possible that the intestinal microbiota is becoming a possible tool to be used in this battle versus obesity. The common forms of obesity are multifactorial and come from a complex interplay of the environmental changes and the individual genetic predisposition. Increasing proof suggests a central role played by the alterations of intestinal microbiota that could represent the causative link between environmental factors and the onset of obesity. The useful effects of intestinal microbiota are mainly intervened by the secretion of various metabolites. In this context, host diet has a direct impact on microbiota composition and is regarded as an important way to modulate the intestinal microbiota.

## 3. Synbiotics, Type of Synbiotics, Synbiotic Action

Gibson and Roberfroid presented the term “synbiotic” to describe a combination of synergistically acting probiotics and prebiotics, the application of which can offer more benefits than the prebiotics or probiotics individually [45]. As the word “synbiotic” comprises a synergy, the term should be restricted to those products in which a prebiotic component selectively favours a probiotic microorganism [46]. Probiotics are effective in the small and large intestine and the influence of a prebiotic is observed, especially in the large intestine. The combination of the two may have a synergistic outcome. The main purpose of that type of combination is the enhancement of survival of probiotic microorganisms in the gastrointestinal tract. Synbiotics have both probiotic and prebiotic features and were created in order to overcome some possible severity in the survival of probiotics in the gastrointestinal tract.

Therefore, a suitable combination of both components in a single product should ensure a better effect, compared to the activity of the probiotic or prebiotic alone [47,48]. Without the participation of prebiotics in food, the supply and use of high doses of commercial probiotics would not be useful and would not produce the secondary metabolites that may carry out the beneficial effects on the host; therefore, it is not surprising that synbiotics have arisen as a spare in order to offer further benefits compared to probiotics or prebiotics alone. Lately, the International Scientific Association for Probiotics and Prebiotics (ISAPP) convened a panel of nutritionists, physiologists, and microbiologists to review the definition and scope of synbiotics. The definition of synbiotic has been updated to “a mixture comprising live microorganisms and substrate(s) selectively utilized by host microorganisms that confers a health benefit on the host” [49].

The following two types of synbiotics were described:

(1) synergistic synbiotics—synbiotics in which the substrate is proposed to be used selectively by the co-administered microorganism(s).
(2) complementary synbiotics—synbiotics composed of a probiotic in combination with a prebiotic, that is intended for targeting the autochthonous microorganisms [49].

In both cases, the influence of synbiotic must be confirmed at the target host and its safety should be validated. The active components of a synbiotic must be adequately stable. Safeguarding the stability of the live microbial component of a synbiotic can be challenging. After live microorganisms are combined with a substrate(s) in a matrix (for example, liquid, dried, or ointment), the load is on producers to ensure that the dose of the live microorganism necessary to confer the stated health advantage is delivered throughout the self-life. Live microorganism viability is highly dependent on the matrix, storage temperature, pH, and oxygen level of the product as follows: for liquid products, the self-life might be as short as 1–2 weeks, for lyophilized or encapsuled products, the self-life might be as long as 2 years. Packaging and warehousing conditions must control the critical factors, such as water activity and temperature, through the production to distribution and consumption.

The choice of an appropriate probiotic and prebiotic exerting a beneficial effect on the host’s health when used separately should be the first aspect to be taken into account when forming a synbiotic formula. Considering a large number of possible combinations, the use of synbiotics for the modulation of gut microbiota in humans seems promising [50].

Synbiotics are used not only for the improved survival of beneficial microorganisms added to diets or feed, but also for the encourage of the proliferation of specific native bacterial strains present in the gastrointestinal tract [51]. The most appropriate approach seems to be to determine the specific abilities that the prebiotic should have in order to have a beneficial effect on the probiotic. The prebiotic should selectively stimulate the growth of microorganisms with a beneficial effect on health while lacking (or limiting) stimulation of other microorganisms.

In synbiotic products, the most popular appears to be the combination of bacteria of the genus *Bifidobacterium* or *Lactobacillus* with fructooligosaccharides (FOS). The most used combinations of probiotics and prebiotics are as follows: bacteria genus *Lactobacillus* + inulin; *Lactobacillus*, *Streptococcus* and *Bifidobacterium* + FOS; bacteria of the genus *Lactobacillus*, *Bifidobacterium*, *Enterococcus* + FOS; *Lactobacillus* and *Bifidobacterium* + oligofructose; bacteria of the genus *Lactobacillus* and *Bifidobacterium* + inulin [52,53]. Prebiotics are mostly used as a selective medium for the probiotic strain’s growth, fermentation, and intestinal passage. Probiotic microorganisms have been reported in the literature as a result of the use of prebiotics, which are more tolerant to the environmental conditions, including oxygenation, pH, and temperature, in the intestines of a particular organism [54]. This combination of ingredients leads to the creation of viable microbiological food supplements, and the provision of a suitable environment allows a positive impact on the health of the host.

Two known modes of synbiotic effects are as follows [55]:

(1) The effects through the improved viability of probiotic microorganisms.
(2) The effects by providing specific actions on health.

The stimulation of probiotics with prebiotics leads to a modulation of metabolic activity in the intestine with preservation of the intestinal biostructure, development of the beneficial microbiota, and the inhibition of potential pathogens present in the gastrointestinal tract. Synbiotics result in reduced concentrations of undesirable metabolites as well as the inactivation of nitrosamines and other cancerogenic substances. Their use leads to a significant increase in the levels of short-chain fatty acids, ketones, carbon disulphides, and methyl acetates, which can have a positive effect on the health of the host [55]. In terms of their therapeutic efficacy, the desirable properties of synbiotics include antibacterial, anticancerogenic, and anti-allergic effects [56,57,58]. They also act against putrefaction in the gut and prevent constipation and diarrhea.

Synbiotics have been shown to be highly effective in preventing osteoporosis, lowering fat and blood sugar levels, regulating the immune system and treating brain disorders associated with abnormal liver function [59]. The concept of synbiotics based on the modification of intestinal microbiota by probiotic microorganisms and appropriately selected prebiotics as their substrates is summarised in Table 2 as the examples of some clinical studies on the effects of synbiotics on obesity and metabolic consequences in children and adults. In obesity, weight adjustment is the most important in both children and adults, as several studies suggest. Anti-obesity (reduced BMI-Z-score, waist circumference) and hypolipidmic effects hypolipidemic effects on triacylglycerides (TAG), and light density lipoprotein cholesterol (LDL-C) of synbiotic supplements in children and adolescents with high BMI have been demonstrated by Safavi et al. [60]. The results of the first study showed the effects of synbiotics on oxidative stress in children and adolescents with primary obesity by altering antropometric parameters, lipid parameters, and serum levels of total oxidative stress [61]. Similarly, overweight and obese adults have experienced significant weight loss, reduced waist circumferences and food intake, and altered intestinal microbiota and their metabolites after six months of synbiotic administration [27,62,63,64,65,66,67,68,69]. The effects of synbiotic supplementation on the body composition parameters and biomarkers of obesity showed an association between a decrease in blood glucose and an increase in the amount of *Lactobacillus* in the synbiotic and placebo groups. In both groups, the mean decrease in HbA1c was accompanied by an average increase in *Lactobacillus* abundance. However, the decrease in body mass, BMI, waist, and body fat over time was associated with a statistically significant decrease in *Bifidobacterium* abundance in both placebo and synbiotic groups [66]. Synbiotic *Bifidobacterium animalis* subsp. lactis 420+Litesse Ultra polydextrose increased the incidence of *Akkermansia*, *Christensenellaceae,* and *Methanobrevibacter*, while the incidence of *Paraprevotella* was reduced. The *Christensenellaceae* family is associated with a poor phenotype, as found in a negative correlation with waist-hip ratio and energy intake, and body fat and cholesterol indicators in the waist-area [27]. Obesity is associated with many changes in the values of physiological parameters, e.g., glucose level, blood pressure, lipid profile, and intestinal microbiota composition of the gut microbiota alone or a combination of several parameters. Several studies revealed that synbiotics have a positive effect on blood glycemic control [70,71,72,73,74,75,76,77,78], lipids [79], blood pressure [80,81,82]. In prediabetic patients, synbiotic treatment improved FPG (Fasting plasma glucose), FIL (fasting insulin levels), HbA1c (glycosylated hemoglobin), insulin resistance, insulin sensitivity, and lipid profile compared with placebo, while probiotics affected only HbA1c. These findings suggest that a combination of probiotics and prebiotics in the synbiotics supplementation synbiotics supplementation is more effective in controlling blood glucose than probiotics alone. In addition, synbiotics led to a greater decrease in HOMA-IR (Homeostatis model assessment of insulin resistance) and an increase in QUICKI (Quantitative insulin sensitivity check index), although there was no difference in microbial levels [83,84,85]. The available human data on the benefits of synbiotic supplementation for metabolic syndrome (MetS) are still limited. Synbiotics, therefore, remain a potentially promising strategy for modulating the intestinal microbiota and treating MetS. Núñez-Sánchez et al. [86] reviewed recent clinical trials in which the effects of synbiotics against various MetS-related parameters were studied. Intestinal microbiome studies revealed that obese and MetS individuals have changes in their intestinal microbiome, including a reduced diversity compared to lean subjects. Synbiotic supplementation aimed at modifying the intestinal microbiome can have a positive effect on weight loss [87]. The anti-obesity mechanism associated with the stabilisation of the intestinal microbiota is thought to involve the secretion of intestinal hormones such as PYY and GLP-1, well-known anorexigenic neurotransmitters that decrease appetite [88]. The study by Rabiei et al. [68] showed that the supplementation with synbiotics composed of different strains of bifidobacteria and lactobacilli plus prebiotic FOS in MetS patients on a weight loss diet was able to delay the plateau phase until week 12, compared to the placebo group that achieved the plateau phase at week six. Weight loss was associated with a decrease in caloric intake and an increase in GLP-1 and PYY levels; however, no significant changes in caloric intake were observed between weeks 6 and 12. Consistent with this study, the supplementation with a synbiotic containing various strains of lactobacilli plus FOS and inulin for 60 days resulted in an improved waist circumference and visceral adiposity index in elderly MetS volunteers [74]. Cardiovascular disease (CVD) is one of the major obesity-related diseases. Synbiotic supplementation in combination with drug therapy is a promising strategy in the prevention and treatment of CVD [89,90,91].

### Synbiotic Functional Foods for Targeting Obesity

Functional food products that affect many physiological functions in the body can be promising treatments for obesity and obesity-related disorders. These naturally processed foods contain bioactive compounds (probiotics and prebiotics) that have been clinically proven to provide health, well-being, and performance benefits beyond normal nutrition alone, thanks to their incorporation into the diet.

The probiotics themselves have the following multiple and diverse effects on the host: (1) antagonistic effects on various microbiota and competitive adhesion to the mucosa and epithelium (antimicrobial activity); (2) causing an increase in mucus production and influencing the barrier integrity (an improved barrier function); (3) can modulate the human immune system (immunomodulation).

Finally, perhaps the most promising approach to the development of effective synbiotic functional foods is the addition of supplementary prebiotic ingredients such as inulin and FOS to probiotic foods [92]. The incorporation of prebiotic functional ingredients into foods exhibits a number of unique physiochemical properties that can potentially: (a) improve the nutritional profile and nutritional value; (b) to improve the texture and sensory properties of foods, in particular due to their volume, to increase their viscosity and to improve the overall sensation in the body and mouth; (c) reduce fat, sugar, and energy without compromising the taste and texture of the product. Synbiotics as the combination of synergistic probiotics and prebiotics may provide greater anti-obesity benefits than prebiotics or probiotics alone [93]. Countless research articles have demonstrated the effectiveness of functional foods as anti-obesity agents through several mechanisms, such as appetite suppression and satiety, decreased lipid absorption, increased thermogenesis, reversed intestinal dysbiosis, and more.

Although the potential of these synbiotics products in the treatment of obesity is further explored, the formation of intestinal microbiota by synbiotics could be a promising new means of preventing and treating obesity and obesity-related disorders, and it can serve as an excellent alternative strategy for developing safe anti-obesity drugs. Functional foods are less likely to have side effects because they are simply common or fortified foods with bioactive compounds. In addition, the availability of functional foods can benefit many individuals of vulnerable populations, such as people from developing countries or those with lower socio-economic status. Despite the proven biological properties of synbiotics, research in this area must focus on the correct selection of probiotic strains, prebiotics, and transport systems in order to avoid suppressing their synergistic or complementary effects on human health. The future direction should lead to the development of functional food products containing stable synbiotics adapted to different age groups or specifically designed to meet the needs of adjuvant therapy. Co-encapsulated synbiotics could be one of the modern approaches currently being developed to modulate the intestinal microbiota, with an emphasis on the health benefits. However, the disadvantage of using synbiotics is that it is difficult to predict the selectivity and specificity of each component and what the resulting mechanism of action will be [94].

## 4. Conclusions

The current obesity epidemic, which is afflicting a large part of society, requires sustainable, affordable, and efficient treatments to combat this public health crisis. Obesity is the result of an energy imbalance involving other factors such as an inadequate lifestyle, brain function, and hormonal mechanisms, as well as genetic and epigenetic factors. This multifactorial pathogenesis may partly explain why obesity is an important challenge for clinical treatment and health policy. Bariatric surgery that reduces weight and controls obesity-related comorbidities is a highly invasive procedure with a high risk of adverse events and poses safety issues in the paediatric population. Thus, it seems clear that the main treatment of obesity should be based on a multicomponent approach involving behaviour therapy, dietary changes, physical activity, and pharmacotherapy. However, the obese subjects expect tangible results in a very short time, which makes the long-term implementation of lifestyle changes difficult. Although the etiology of obesity is multifactorial and incredibly complex, several studies have shown the potential therapeutic effects of synbiotics on body weight, BMI, waist circumference, fat deposition, lipid profile, and chronic inflammation state. Recent research strongly implicates intestinal dysbiosis as a key contributor to the development of obesity and related metabolic abnormalities. New approaches in the treatment and prevention of obesity and related metabolic disorders are necessary. Dosage, duration of treatment, and long-term effects of the administration of different microbial strains are still under investigation; more studies are needed to rationally prescribe probiotics to prevent or treat obesity. Diet control, as well as the environmental and lifestyle factors that support the development of obesity, remains the best solution to the problems associated with weight gain. Consequently, the modulation of the intestinal microbiota to restore a stable, coherent metabolic state has become a research area of great interest in recent years. Further research, including well-controlled, randomized, and controlled clinical trials, is needed to better understand the mechanisms involved in the anti-obesogenic effects of synbiotics in order to develop safe strategies for obesity prevention and management.

## Figures and Tables

**Table 1:** Major bacteria in the human gut microbiota.

Domain	Phylum	Class	Order	Family	Genus
***Bacteria***	*Bacteridetes*	Bacteroidia	Bacteroidales	Bacteroidacee	Bacteroides
				Prevotellacee	Prevotella
					Xylanibacter
				Rikenellacee	Rikenella
	*Firmicutes*	Clostridia	Clostridiales	Clostridiacee	Clostridium
				Ruminococcae	Faecalibacterium
					Ruminococcus
				Peptostreptococcae	Peptostreptococcus
					Fusibacter
				Eubacteriacee	Eubacterium
				Veillonellacee	Veillonella
				Lachnospiraceae	Roseburia
		Bacilli	Bacillales	Bacillaceae	Bacillus
				Lysteriaceae	Lysteria
				Staphylococcaceae	Staphylococcus
				Pasteuriaceae	Pasteuria
			Lactobacillales	Lactobacillaceae	Lactobacillus
				Enterococcaceae	Enterococcus
				Streptococcaceae	Streptococcus
	*Actinobacteria*	Actinobacteria	Bifidobacteriales	Bifidobacteriaceae	Bifidobacterium
					Gardnerella
			Actinomycetales	Actinomycetaceae	Actinomyces
	*Proteobacteria*	Deltaproteobacteria	Desulfobacteriales	Desulfobulbaceae	Desulfovibrio
		Gammaproteobacteria	Enterobacteriales	Enterobacteriaceae	Escherichia
					Enterobacter
					Klebsiela
					Proteus
		Epsilonproteobacteria	Campylobacteriales	Campylobacteriaceae	Campylobacter
				Helycobacteriaceae	Helycobacter
	*Fusobacteria*	Fusobacteria	Fusobacteriales	Fusobacteriaceae	Fusobacterium
	*Verrucomicrobia*	Verrucomicrobiae	Verrucomicrobiales	Verrucomicrobiaceae	Verrucomicrobium
	*Synergistetes*	Synergistia	Synergistales	Synergistaceae	Synergistes
	*Spirochaetes*	Spirochaetes	Spirochaetales	Spirochaetaceae	Spirochaeta
					Treponema
	*Cyanobacteria*	Cyanobacteria			

**Table 2:** Clinical trials concerning the effect of synbiotics on obesity and obesity related metabolic disorders in children and adults.

References	Subjects	Synbiotic Composition	Duration of Administration	Major Outcome
[27]	134 overweight or obese participants	*B. animalis +* Litesse Ultra polydextrose	6 months	Decreased weight, decreased plasma bile acids, altered of the gut microbiota increased *Akkermansia*, *Christensenellaceae, Methanobrevibacter*, improved intestinal barrier function and markers associated with obesity
[62]	225 obese volunteers	*B. animalis* + Litesse Ultra polydextrose	6 months	Reduced waist circumference and food intake
[63]	153 obese women and men	*L. rhamnosus* CGMCC1.3724 + oligofructose and inulin	24 weeks	Lose weight and fat mass, reduced leptin level, increased of *Lachnospiraceae* in faeces
[60]	70 children and adolescents with elevate BMI	*L. casei*, *L. rhamnosus*, *S. thermophilus*, *B. breve*, *L. acidophilus*, *B. longum*, *L. bulgaricus* + FOS	8 weeks	Decreased in BMI Z-score and waist circumference, increased waist-to-hip ratio, significant decreased TAG and LDL-C
[61]	77 children and adolescent with primary obesity	*L. acidophilus*, *L. rhamnosus*, *B. bifidum*, *B. longum*, *E. faecium* + FOS	4 weeks	Significant reduction of weight and BMI, decreased TC, LDL-C, and total oxidative stress serum levels
[70]	38 subjects with overweight or obesity or metabolic syndrome	*L. casei*, *L. rhamnosus*, *S. thermophilus*, *B. breve*, *L. acidophilus*, *B. longum*, *L. bulgaricus *+ FOS	28 weeks	Significantly improved the levels of fasting blood sugar and insulin resistance
[71]	54 patients with T2D	*L. acidophilus*, *L. casei*, *L. rhamnosus*, *L. bulgaricus*, *B. breve*, *B. longum*, *S.* + FOS	8 weeks	Decreased FPG, increased HOMA-IR, elevated GSH in plasma, reduced serum hs-CRP
[72]	62 patients with T2D	*L. sporogenes +* inulin	6 weeks	Significantly decreased insulin level, HOMA-IR, hs-CRP, increased levels of lipid profile (TC, LDL-C, TAG, HDL-C), increased plasma total GSH and uric acid levels
[73]	81 patients with T2D	*L. sporogenes* + inulin	8 weeks	Significantly reduced insulin level, HOMA-IR, and homeostasis model assessment b cell function (HOMA-B)
[74]	60 overweight T2D patients with CHD	*L. acidophilus*, *L. casei*, *B. bifidum* + inulin	12 weeks	Significantly decreased fasting plasma glucose, serum insulin concentration, HOMA-B, increased QUICKI, changed HDL-C level
[79]	78 patients with T2D	*L. sporogenes* + inulin	8 weeks	Decreased lipid profile (TAG, TC/HDL-C) and significant elevated HDL-C level
[75]	30 patients with T2D	*L. acidophilus*, *B. bifidum* + oligofructose	2 weeks	Elevated HDL-C level, reduced fasting glycaemia, reduction of TC, TAG
[80]	40 subjects with metabolic syndrome	*L. casei, L. rhamnosus*, *S. thermophilus*, *B. breve*, *L. acidophilus*, *B. longum*, *L. bulgaricus* + FOS	12 weeks	Synbiotics had synergistic effects on improving systolic blood pressure and anthropometric measurements
[68]	46 subjects with metabolic syndrome	*L. casei*, *L. rhamnosus*, *S. thermophilus*, *B. breve*, *L. acidophilus*, *B. longum*, *L. bulgaricus* + FOS	12 weeks	Decreased body weight, BMI, FBS, HOMA-IR, increased GLP-1, PYY
[77]	120 patients with T2D	*Bacillus coagulant*, lactic acid + β–glucan, inulin	12 weeks	Decreased HbA1c, increased SOD
[78]	115 patients with T2D	*L. acidophilus* + cinnamon	12 weeks	Decreased FPG, HbA1c and advanced glycation end products
[83]	120 prediabetic patients	*L. acidophilus*, *B. bifidum*, *B. lactis*, *B. longum*, + inulin	24 weeks	Decreased FIL, HOMA-IR, HbA1c, FPG, increased QUICKI
[81]	120 prediabetic patients	*L. acidophilus*, *B. bifidum*, *B. lactis*, *B. longum,* + inulin	24 weeks	Decreased metabolic syndrome prevalence, obesity, hyperglycemia, hypertension, HDL-C
[84]	120 prediabetic patients	*L. acidophilus*, *B. bifidum*, *B. lactis*, *B. longum*, + inulin	24 weeks	Decreased TAG,
[85]	120 prediabetic patients	*L. acidophilus*, *B. bifidum*, *B. lactis*, *B. longum*, + inulin	24 weeks	Synbiotic had no significant effect on the changes in bacteria, in probiotic group was increased ratio *Bacteroides fragilis* to *Escherichia coli*, decreased Firmicutes to Bacteroidetes ratio
[76]	60 diabetic patients undergoing hemodialysis	*B. bifidum*, *L. acidophilus*, *L. casei* + inulin	12 weeks	Decreased FPG, FIL, HOMA-IR, HbA1c, increased QUICKI, reduced hs-CRP, MDA, increased TAC, GSH
[64]	60 overweight or obese adults	*L. acidophilus*, *L. casei*, *B. bifidum* + inulin	8 weeks	Reduced body weight, TC, TAG, LDL-C, stress anxiety
[65]	60 military personnel with metabolic syndrome	*L. casei*, *L. acidophilus*, *L. rhamnosus*, *L. bulgaricus*, *B. breve*, *B. longum*, *S. thermophiles* + FOS	8 weeks	Decreased BMI, FPG, TAG
[66]	20 healthy overweight volunteers	*L. acidophilus*, *B. lactis*, *B. longum UABl-14*, *B. bifidum* + trans-galactooligosaccharide mixture	3 months	Decreased body mass, BMI, waist circumference, body fat mass, HbA1c, changed of intestinal microbiota-increased *Bifidobacterium, Lactobacillus, Ruminococcus, Verrucomicrobiae*
[82]	60 elderly patients with metabolic syndrome	*L. plantarum*, *L. acidophilus*, *L. reuteri* + inulin, FOS	60 days	Improvement in visceral adiposity, decreased waist circumference, TC, HDL, TAG, hs-CRP, TNF-α, decrease metabolic syndrome prevalence and MAP
[69]	41 healthy sedentary overweight and obese volunteers	*Bacillus coagulant* + β-glucans	12 weeks	Decreased hs-CRP, LDL/HDL, resistin, lower *Bifidobacterium* spp., increased *Faecalibacterium prausnitzii*
[67]	40 subjects with obesity	*L. plantarum*, *S. thermophilus*, *B. bifidum* + FOS	8 weeks	Significantly decreased FBG, body weight and BMI was not reduced

Abbreviation: BMI—body mass index, FOS—fructooligosaccharides, T2D—type 2 diabetes mellitus, HOMA-IR—homeostasis model assessment of insulin resistance, HOMA-B—homeostasis model assessment b cell function, TC—total cholesterol, LDL-C—light density lipoprotein cholesterol, TAG—triacylglycerides, HDL-C—high density lipoprotein cholesterol, GSH—glutathione, hs-CRP—high sensitivity C-reactive protein, HbA1c—glycosylated hemoglobin, SOD—superoxid dismutase, FPG—fasting plasma glucose, FIL—fasting insulin levels, FBG—fasting blood glucose, QUICKI—quantitative insulin sensitivity check index, MDA—malondialdehyde, TAC—total antioxidant capacity, GLP-1—glucagon-like peptide-1, PYY—peptide YY, MAP—mean arterial pressure, TNF α—tumor necrosis alpha, FBS—fasting blood sugar.
